# Structuring healthcare advance directives: Evidence from Chinese end‐of‐life cancer patients' treatment preferences

**DOI:** 10.1111/hex.13769

**Published:** 2023-04-27

**Authors:** Zi‐Meng Ye, Ben Ma, Elizabeth Maitland, Stephen Nicholas, Jian Wang, An‐Li Leng

**Affiliations:** ^1^ School of Political Science and Public Administration Shandong University Qingdao China; ^2^ School of Management University of Liverpool Liverpool UK; ^3^ Australian National Institute of Management and Commerce Eveleigh New South Wales Australia; ^4^ Newcastle Business School University of Newcastle Newcastle New South Wales Australia; ^5^ Dong Fureng Institute of Economic and Social Development Wuhan University Beijing China

**Keywords:** advanced cancer, default options, health care, order effect, treatment preferences

## Abstract

**Background:**

Patients' treatment decisions may be influenced by the ways in which treatment options are presented. There is little evidence on how patients with advanced cancer choose preferences for advance directives (ADs) in China. Informed by behavioural economics, we assess whether end‐of‐life (EOL) cancer patients held deep‐seated preferences for their health care and whether default options and order effects influenced their decision‐making.

**Methods:**

We collected data on 179 advanced cancer patients who were randomly assigned to complete one of the four types of ADs: comfort‐oriented care (CC) AD (comfort default AD); a life extension (LE)‐oriented care option (LE default AD); CC (standard CC AD) and LE‐oriented (standard LE AD). Analysis of variance test was used.

**Results:**

In terms of the general goal of care, 32.6% of patients in the comfort default AD group retained the comfort‐oriented choice, twice as many as in the standard CC group without default options. Order effect was significant in only two individual‐specific palliative care choices. Most patients (65.9%) appointed their children to make EOL care decisions, but patients choosing the CC goal were twice as likely to ask their family members to adhere to their choices than patients who chose the LE goal.

**Conclusion:**

Patients with advanced cancer did not hold deep‐seated preferences for EOL care. Default options shaped decisions between CC and LE‐oriented care. Order effect only shaped decisions in some specific treatment targets. The structure of ADs matters and influence different treatment outcomes, including the role of palliative care.

**Patient or Public Contribution:**

Between August and November 2018, from 640 cancer hospital medical records fitting the selection criteria at a 3A level hospital in Shandong Province, we randomly selected 188 terminal EOL advanced cancer patients using a random generator programme to ensure all eligible patients had an equal chance of selection. Each respondent completes one of the four AD surveys. While respondents might require support in making their healthcare choices, they were informed about the purpose of our research study, and that their survey choices would not affect their actual treatment plan. Patients who did not agree to participate were not surveyed.

## 1 INTRODUCTION

Between 70% and 90% of Chinese advanced cancer patients are hospitalized and implicitly receive life‐extension (LE) treatments during the last 3 months of their life.^1^ Routine life‐extending hospital treatments frequently involve severe pain, which is especially controversial when advanced cancer patients in interviews prefer comfort and dignity over LE interventions.^2^ Previous studies found that treatment decisions are not made solely by the advanced cancer patient, but made, or influenced, by family caregivers, who had a greater willingness than patients themselves to choose life‐extending treatments.^3^ The view of life and death in Chinese traditional culture advocates the view of letting nature take its course, hoping that patients themselves can be understood and respected, and maintain their dignity of life.^4^ Advance directives (ADs) enable patients to express their healthcare preferences, including no treatment or active interventions, before starting their end‐of‐life (EOL) care when they may not be able to communicate their treatment wishes.^5^, ^6^, ^7^, ^8^ ADs are tools or instruments that aim to ensure patients' wishes and preferences are respected, but ADs themselves do not necessarily ‘respect the care wishes’ of patients because patients' care wishes may not be clear or are overridden by family caregivers or doctors. In some countries, such as America, Canada, England, Spain, German and France, countries' national policies and practices encourage the completion of ADs.^9^, ^10^, ^11^, ^12^ Degenholtz et al. and Silveira et al. found that American elderly patients who completed ADs were less likely to die in nursing homes or hospitals and more often receive care consistent with their preferences.^13^, ^14^ Halpern et al. found that ADs provide seriously ill US patients with an opportunity to counter the tendency to administer treatments to extend life.^15^

Given the importance of treatment choices embedded in ADs, it is important to understand how the structure of ADs might affect patient preferences. Previous studies found that using default options, or an already checked treatment choice, shaped patients' choices, and it follows that patients might change their choices with different, or no, default options.^15^ Default options studies further suggest that people may not have well‐articulated and firm views about the types of care that best promote their values at the EOL.^16^ By being easily influenced by default options, seriously ill patients may express no clear preference for, say, hospice care or invasive treatment. Behavioural economics suggests that the preference for hospice care or invasive treatment might be ‘constructed’ when people are asked to select from a predetermined list of options, rather than reflecting a patient's deep‐rooted preferences.^17^

Although the effect of the default option on patients has been verified in several studies,^5^, ^6^, ^7^, ^8^, ^13^, ^14^, ^15^ its effect on patients with advanced cancer remains unstudied. Unlike other types of seriously ill patients, patients with advanced cancer are in an irreversible and painful healthcare stage and EOL cancer care can only delay death for a short period of time. For patients receiving active treatment, life is usually only extended for 1 or 2 years, with little hope of short‐term improvement and no hope of long‐term recovery.^18^ We conducted a survey to determine whether default options influenced the ADs choices of a large sample of Chinese advanced cancer patients.^15^ Second, we set up two standard AD groups to verify the position‐dependent order effects, where the order of options may affect the choice of options given that the first option presented usually dominates.^19^ In addition to testing patients about their general goals for healthcare, we also examine cancer patient preferences for 11 specific forms of treatment, including traditional Chinese medicine treatment and palliative care. This study provides evidence on how patients with advanced cancer select preferences for ADs in China; identify whether advanced cancer patients held deep‐seated preferences for their EOL care and tests whether default options and the order effect impacted patient decision‐making. If patients' decision‐making were influenced by the default options or order effect, it suggests that patients' preferences are not deep‐rooted and can be changed.

## 2 METHODS

### 2.1 Study design

We conducted a four‐group AD survey in Chinese to test for changes in decision‐making with default options and with different option orders. Following Halpern et al.,^15^ we set up two standard AD groups to verify the influence of the default and order effect on ADs choices. To test patients about their general healthcare goals, we examined EOL cancer patients' preferences for 11 specific treatments. Cancer patients completed a survey with different random default options for healthcare treatment preferences to receive eight forms of LE support treatments (cardiopulmonary resuscitation [CPR], intensive care unit [ICU] admission, mechanical ventilation, dialysis, surgical feeding tube, surgery, radiation and chemotherapy), two forms of palliative care (palliative care which costs US$1059 per month, palliative care which costs US$454 per month) and traditional Chinese medicine treatment. Palliative care is a comfort care approach that improves the quality of life of patients and relieves suffering through the treatment of pain and other problems. The role of traditional Chinese medicine as a traditional Chinese treatment for cancer is still debatable,^20^ but the pain produced in the process is far less than the first eight treatments, so it is ‘comfort’. The difference in price between the two palliative treatments is to test the willingness of cancer patients to pay for palliative treatment. Copies of the ADs survey can be found in the online Supporting Information: 1. We also collected information on respondents' background characteristics, comprising sex, age, cancer type and cancer stage and information on the respondents' family members when the patient was unable to make their own healthcare decisions without support. While respondents might require support in making their healthcare choices, they were informed about the purpose of our research study, and that their survey choices would not affect their actual treatment plan. Patients who did not agree to participate were not surveyed.

### 2.2 Sample

Four trained students from the School of Public Health at Shandong University randomly selected cancer patients from the hospital information system at a 3A level hospital in Shandong Province in China. The students were trained in interview techniques and research protocols. The selection criteria were aged over 40 years old; diagnosed with stage III or IV cancer; hospitalized and receiving active treatment (such as surgery, radiotherapy, chemotherapy, targeted therapy) and without cognitive impairments.

With the help of medical staff, our investigators met the patients or their families to explain the purpose and significance of the study, discuss confidentiality and data security and receive written informed consent. The trained students then conducted the face‐to‐face questionnaire interviews. Each respondent received a cash reward of 50 yuan after completing the survey. The study was approved by Shandong University Ethics Committee.

Between August and November 2018, from 640 cancer hospital medical records fitting the selection criteria at a 3A level hospital in Shandong Province, we randomly selected 188 terminal EOL advanced cancer patients using a random generator programme to ensure all eligible patients had an equal chance of selection. Five patients did not participate in the survey, leaving a sample of 183 patients and a 97.3% response rate. Finally, 179 patients completed the survey.

### 2.3 Randomization and interventions

Using the randomly generated list of participants, we allocated the four ADs in repetitive order to respondents. Each respondent complete one of the four ADs surveys: a comfort‐oriented care (CC) AD (comfort default AD), where comfort care was put first in the list of all EOL care choices and preselected by default; a LE‐oriented care option (LE default AD) where the LE option was put first in the list of all EOL care options and preselected by default; CC (standard CC AD) choice where CC AD was put first in the list of all EOL care options, but no choices were preselected and LE oriented (standard LE AD) care option where LE AD was put first in the list of all EOL care options, but no choices were preselected (Supporting Information 2: eTable 1). The comfort default AD and the LE default AD were identical, except the comfort‐oriented (or LE) goals went listed first and were preselected. The preselected option was clearly marked with an ‘X’. Respondents were informed by research staff and written instructions that other choices could be made by crossing out the preselected option and selecting alternative treatment options, which were identical to those in the standard CC AD and standard LE AD groups. To study the order effects, respondents in the two standard groups received two versions with either comfort or life‐extending‐oriented care options listed first, but not preselected. All patients were asked to choose an overall care goal and 11 specific care objectives with eight forms of life support interventions, two forms of palliative care and a traditional Chinese medicine treatment listed. Patients could expand upon or clarify their choices by writing in the additional space provided in every survey version. As shown in online Supporting Information: 1, there were four groups with a total of four survey versions. The design of the survey was informed by William Knaus' prognoses and preferences for outcomes and risks of treatments.^21^ We developed the AD forms for this research. When the eligible patients agreed to participate in the survey, one of four versions of ADs was provided to patients randomly.

### 2.4 Outcomes

Given the propensity of the healthcare system to try to extend life in the absence of an alternative directive, one key outcome was the proportion of cancer patients across the four AD groups who selected a comfort‐oriented healthcare goal versus the LE goal. Secondary outcomes included the choices cancer patients made in 11 specific treatment options.

### 2.5 Statistical analysis

Our analysis assessed the efficacy of default options in ADs among cancer patients who completed the questionnaire. Data analysis was conducted using Stata version 15. Statistical significance was set at *p* < .05. The noninferiority tests were analysis of variance (ANOVA).

### 2.6 Patient and public involvement

No patients were actively involved in setting the research question, or outcome measures nor involved in the design of the study. Patients were not involved in the interpretation or write‐up of the results, nor are there plans for the results to be disseminated to the patient community affected by this research.

## 3 RESULTS

### 3.1 Study sample

The characteristics of the 179 patients are shown in Table 1. Patients were on average 61 years old, mostly males (64%) and married (90%). According to the International Statistical Classification of Diseases and Related Health Problems, 10th Revision, among the 179 patients, 53.6% of patients were diagnosed with urological cancer (renal, bladder and prostatic), 19.7% with digestive system cancer (gastric, colorectal and liver cancer) and 16.4% with lung cancer. As shown in Table 2 (and for specific significance online Supporting Information 2: eTable 1), there were no significant differences between the four AD groups.

**Table 1:** Characteristics of the study sample (*n* = 179).

Characteristics	No. (%)				*p* value
	Comfort default AD (*n* = 43)	LE default AD (*n* = 50)	Standard CC AD (*n* = 43)	Standard LE AD (*n* = 43)	
Age, mean (SD)	63.1 (0.4)	60.5 (0.5)	62.0 (0.5)	63.1 (0.5)	.31
Sex					.70
Male	32 (74.3)	32 (64.0)	31 (72.1)	31 (72.1)	
Female	11 (25.6)	18 (32.0)	12 (27.9)	12 (27.9)	
Marital status					.79
Married	40 (93.0)	45 (90.0)	41 (95.4)	42 (97.6)	
Unmarried/widowed/divorced	3 (7.0)	4 (10.0）	2 (4.6)	1 (2.4)	
Cancer stage					.14
Stage III	27 (62.8)	20 (40.0)	20 (46.5)	24 (55.8)	
Stage IV	16 (37.2)	30 (60.0)	23 (53.5)	19 (44.2)	

Abbreviations: AD, advance directive; CC, comfort‐oriented care; LE, life extension.

**Table 2:** EOL patients' choice of the family member (*n* = 179).

	No. (%)			
	Comfort default AD (*n* = 43)	Standard CC AD (*n* = 43)	LE default AD (*n* = 50)	Standard LE AD (*n* = 43)
Relation				
Spouse	11 (25.6)	10 (23.3)	15 (30.0)	11 (25.6)
Child	28 (65.1)	31 (72.1)	28 (56.0)	31 (72.1)
Parent	2 (4.7)	0 (0)	3 (6.0)	0 (0)
Brothers and sisters	0 (0)	4 (2.3)	1 (2.0)	0 (0)
None	2 (4.7)	1 (2.3)	3 (6.0)	1 (2.3)
Family member power				
Must follow patient's decision	13 (30.2)	6 (14.0)	3 (6.0)	7 (16.3)
Have the right to decide	29 (67.4)	36 (83.7)	45 (90.0)	35 (81.4)
Not selected	1 (2.3)	1 (2.3)	2 (4.0)	1 (2.3)

Abbreviations: AD, advance directive; CC, comfort‐oriented care; EOL, end‐of‐life; LE, life extension.

### 3.2 General healthcare goals

**Figure 1:**
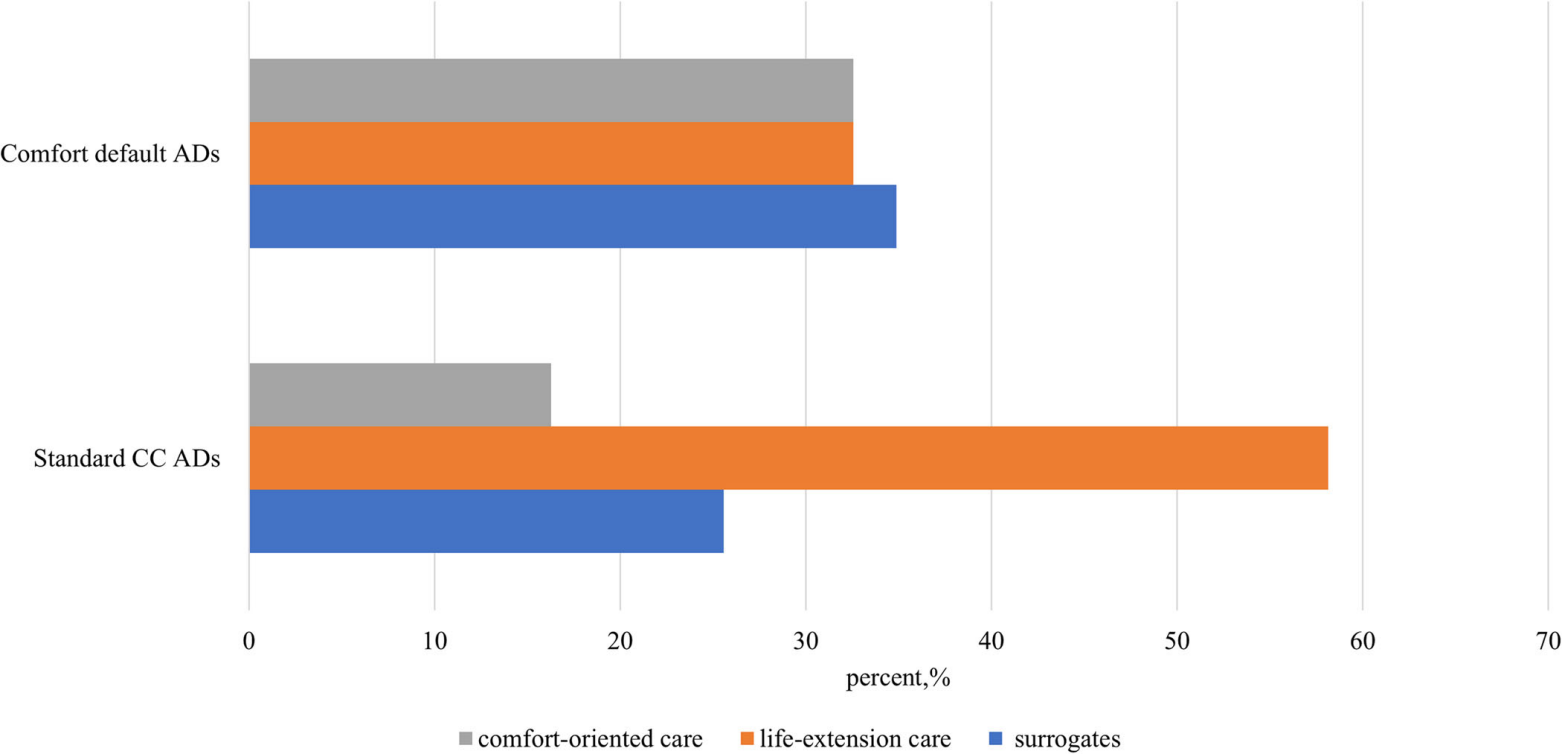
Percentage of cancer patients choosing a general goal of care in comfort default ADs. AD, advance directive; CC, comfort‐oriented care.

**Figure 2:**
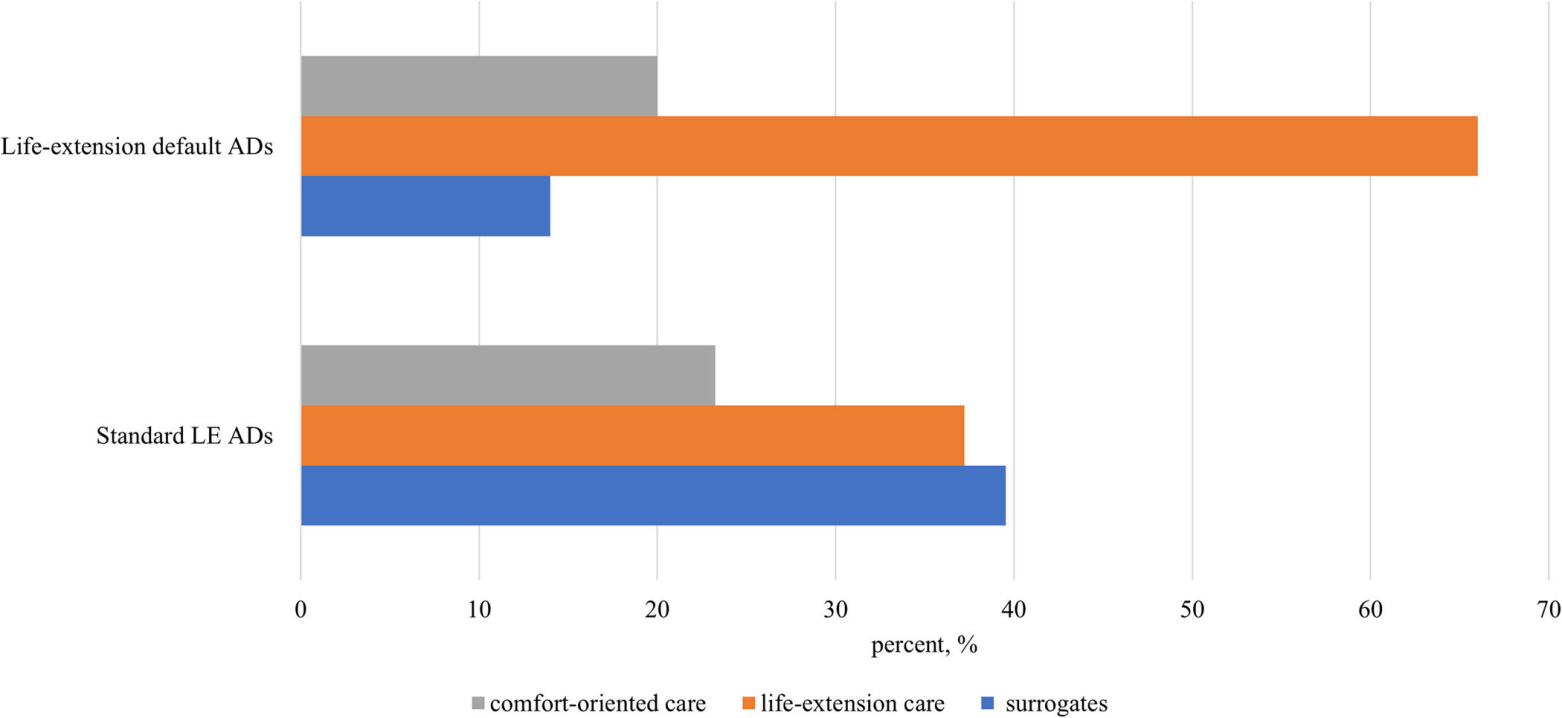
Percentage of cancer patients choosing a general goal of care in LE default ADs. AD, advance directive; LE, life extension.

As shown in Figures 1 and 2, the default options had a significant impact on patients' preferences when comparing the default AD groups and standard AD groups. Specifically, 32.6% of patients in the comfort default ADs group retained the comfort‐oriented choice, twice the standard CC without a default option (16.3%). Second, 66% of patients in the LE default AD group retained the LE choice, which was much higher than those in the standard LE AD group without a default option (37.2%). When making the comfort‐oriented choice, the default option significantly reduced the percentage of patients who choose LE treatments and 32.6% of patients chose the LE care in the comfort default AD group, which was much lower than those in the LE default AD group (66.0%), standard CC AD group (58.1%) and standard LE AD group (37.2%). (*p* = .00 < 0.01; online ANOVA test Supporting Information 2: eTable 3). Based on the default option choice outcomes, our results show that Chinese cancer patients did not hold strong preferences for EOL treatments. The default options significantly affect the choices of patients, which shows that Chinese patients with advanced cancer had no deep treatment preferences.

**Figure 3:**
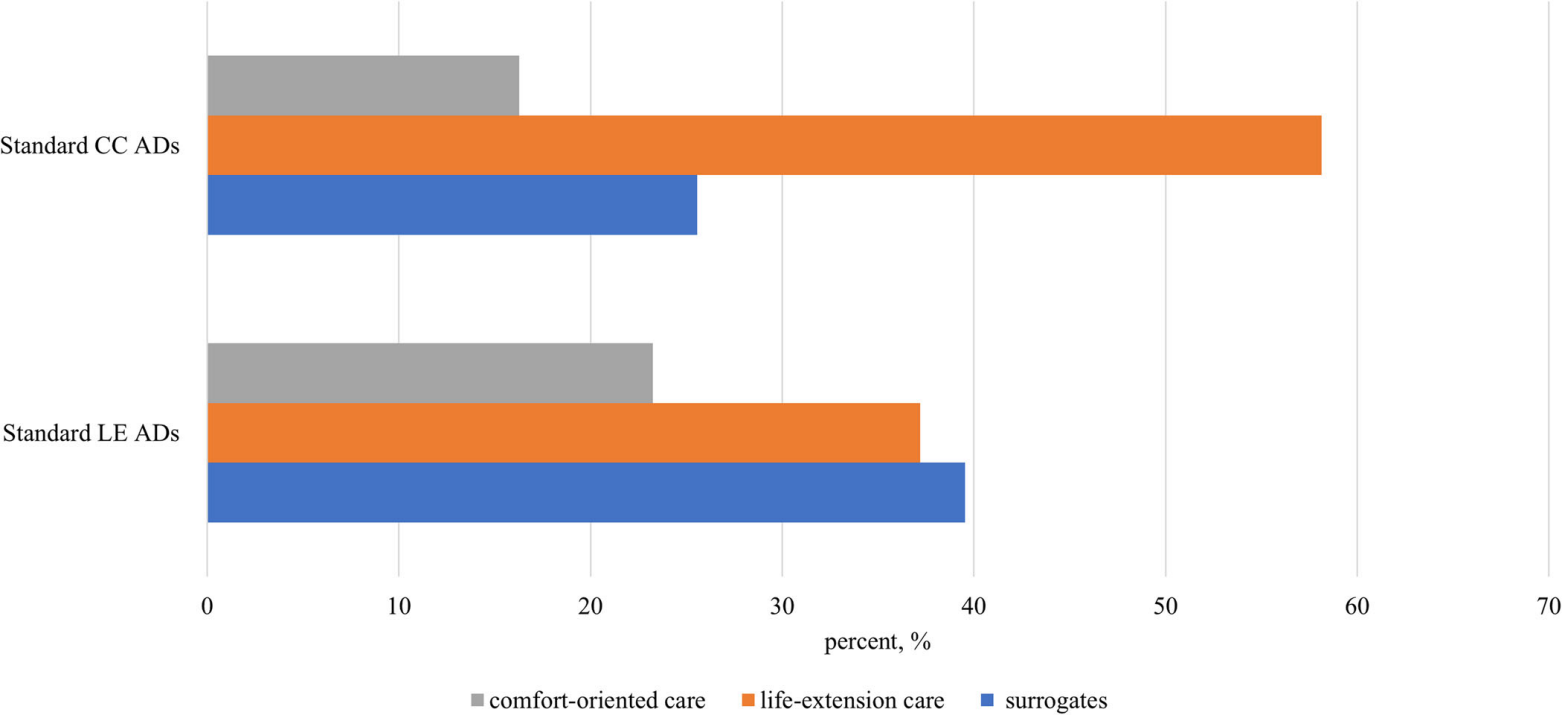
Percentage of cancer patients choosing the general goal of care in different order ADs. AD, advance directive; CC, comfort‐oriented care; LE, life extension.

Second, we tested the position‐dependent order effect of treatment options in Figure 3. We hypothesized an order effect on patients' choices, where more patients would choose the choice option when put in the first place. We found that the order effect did not influence the proportions of choosing general goals of care. To be specific, CC was put in the first place in the standard CC AD group, but the proportion of patients who chose CC was 16.3%, much lower than that of LE care (58.1%). Similarly, although LE care was put in the first place in the standard LE AD group, the proportion of patients who chose LE care was not the highest treatment option. This shows that the order effect did not dominate ADs, and it did not influence the choice of patients with advanced cancer.

### 3.3 Specific objectives of care

**Figure 4:**
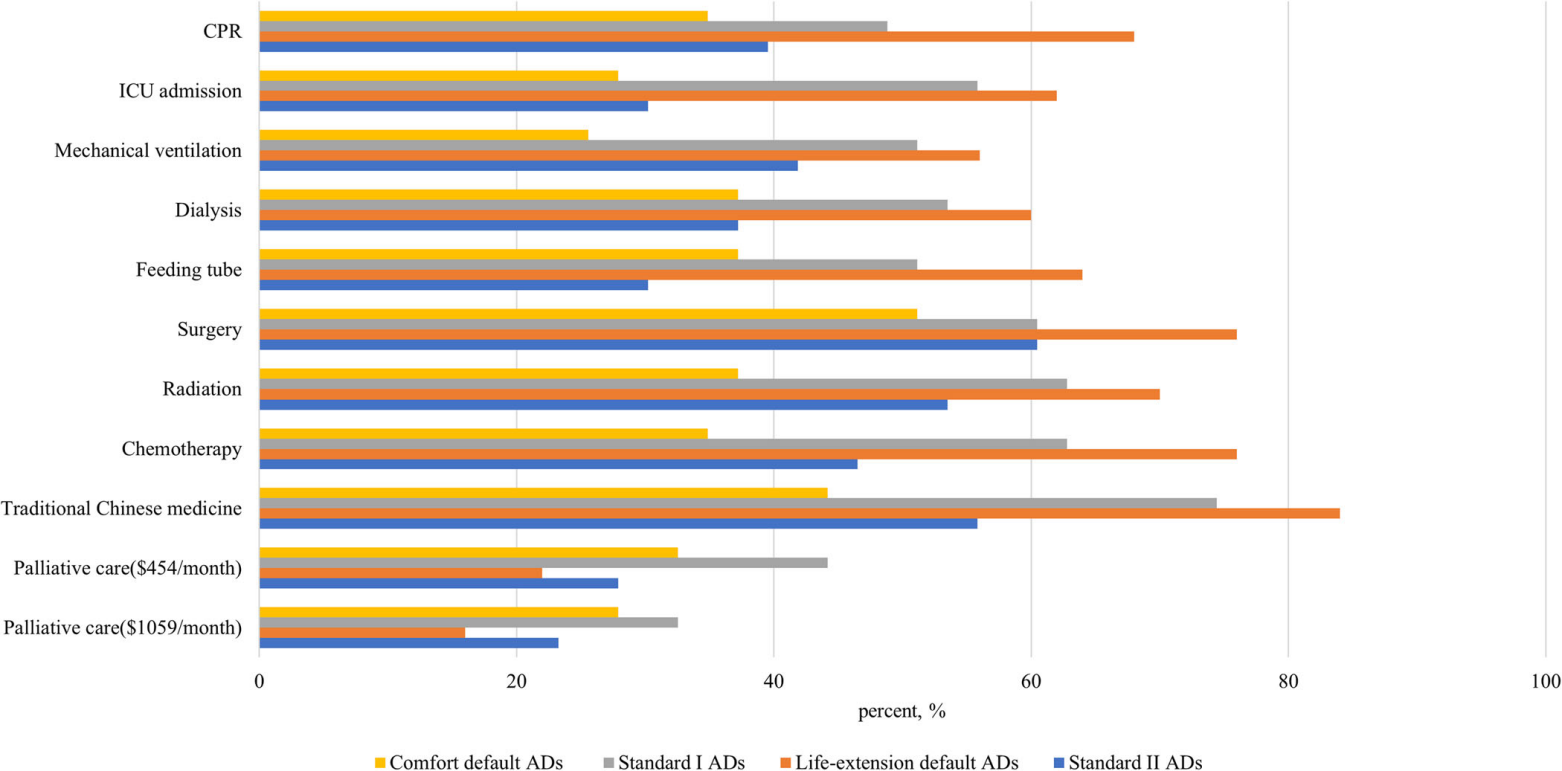
Percentage of cancer patients choosing to accept each treatment (*n* = 179). AD, advance directive; CPR, cardiopulmonary resuscitation; ICU, intensive care unit.

Figure 4 shows that the default options had a significant impact on patients' preferences for specific EOL care. The proportions of patients choosing to accept life support treatments (CPR, ICU admission, mechanical ventilation, dialysis, surgery, radiation, chemotherapy) and Chinese medicine treatment were lowest in the comfort default AD group (*p* < .05, online Supporting Information 2: eTable 3). In contrast, the proportions of patients choosing to accept eight life support treatments and the Chinese medicine treatment were highest in the LE default AD group (*p* < .05, online Supporting Information 2: eTable 3). Further, the proportions of patients choosing to accept two palliative care items (palliative care $454/month and palliative care $1059/month) in the LE default AD group were lower than those in the standard LE AD group.

Figure 4 also shows that the order effect did not impact the eight life support treatments and the Chinese medicine treatment, but did impact the two palliative care items. When palliative care was put in the first order in the standard CC AD group, more patients choose palliative care in the standard CC AD group than the standard LE AD group. The willingness of cancer patients to pay for palliative care did not vary with the price.

### 3.4 Patients' expectations about their family members

EOL cancer patients were also asked about their choice of family member to determine their healthcare when they could no longer make care decisions themselves. As shown in Table 2, the majority of EOL cancer patients (65.9% of 118 patients and more than 50% in each group) chose to appoint their children to make health and personal care decisions when they lack sufficient capacity to make or communicate those choices. Next, EOL patients (26.3%) entrusted their spouses to make decisions, with very few cancer patients selecting their parents (2.8%) or siblings (1.1%). We use the noninferiority tests (ANOVA, *p* = .31) showed that there was no significant difference in the age of patients in the four groups. Among the patients, the oldest was 91 years old and the youngest was 44 years old. Random grouping logistic regression (*p* = .067) and correlation test (*p* = −.13) were used to verify that there was no relationship between family member selection and patient age. There were no statistically significant differences between the four AD groups (*p* > .05, online Supporting Information 2: eTable 4).

**Figure 5:**
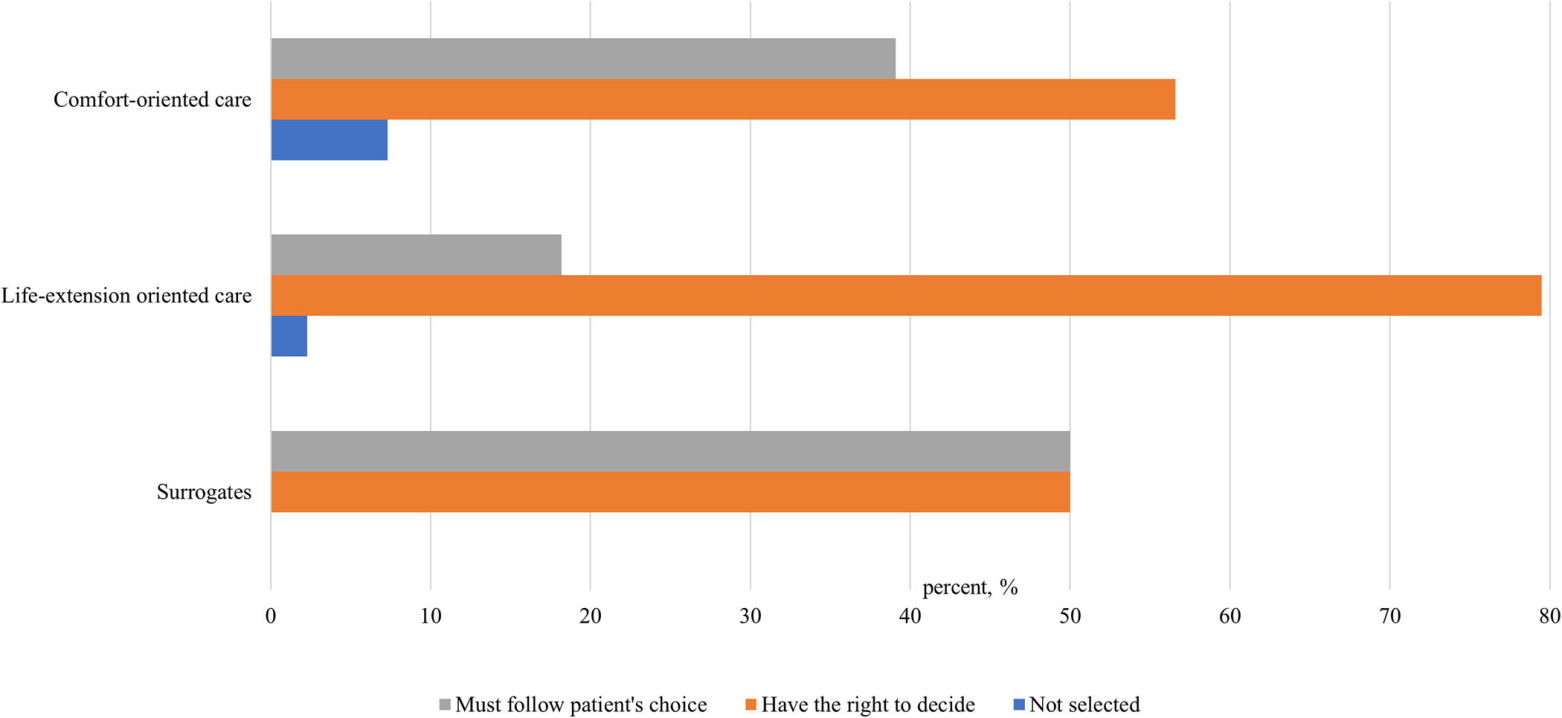
Percentage of cancer patients' agent power among three goals (*n* = 179).

In terms of family member power, 30.2% of patients in the comfort default AD group thought their family member must follow their AD instructions, significantly higher than those in the LE default AD group (6%). Ninety percent (90.0%) of patients in the LE default AD group thought their family member should have the final say in EOL care treatments, much higher than those in the comfort default AD group (67.4%). The differences were statistically significant between the four AD groups (*p* < .05, online Supporting Information 2: eTable 4). Figure 5 assesses whether EOL cancer patients expected their family members to follow or change their healthcare preferences. Taken as a whole, most LE advanced cancer patients (79.5%) expected their family members to exercise some flexibility in terms of adhering to their specific treatment choices. Although patients determine their treatment goal for LE at the beginning of care, they were also willing to make concessions during treatment to reduce any burden on their families. The family member; will decide whether to adhere to or adjust their nursing goal, even if the family member's decision may be contrary to their nursing goal. Only 56.6% of those chose the CC goal. More importantly, about 40.0% of patients who chose the CC goal asked their family members to adhere to their choices, twice that of patients who chose the LE care goal. This shows that patients who choose the CC goal prefer their family members to follow their wishes and maintain personal comfort and dignity in follow‐up treatment than patients who choose extended life care goals.

## 4 DISCUSSION

Our study provides new evidence on how Chinese EOL advanced cancer patients made their AD healthcare preferences and examines the influence of default options and the position‐dependent order effect of treatment options on their decision‐making. Based on the default option choice outcomes, our results show that Chinese cancer patients did not hold strong preferences for EOL care. The default options significantly affected patients’ preferences for EOL care, not only the general goal of care but also the specific care treatment objectives. In our study, the order effect was significant only in the specific objectives of palliative care. Most patients chose to appoint their children to make EOL care decisions and the majority of patients let their family members have flexibility in following their AD preferences when they were unable to make their own healthcare decisions.

Default options have been shown to influence decisions in domains as diverse as drivers' insurance,^18^ retirement savings,^22^, ^23^ influenza vaccination,^24^ and organ donation.^25^ When people have strong preferences, their choices are not affected by the default options or counter the default options. However, when people do not have strong preferences, they are more likely to select the default option.^15^ Our study showed that default options had a significant influence on cancer patients’ preferences for EOL care interventions, not only the general goal of care but also the specific treatment objectives. The comfort‐oriented default options increased the willingness to accept comfort‐oriented EOL care and the LE‐oriented default options significantly increased the willingness to accept LE care.

Considering comfort‐oriented patients had higher levels of emotional stability, positivity, and agreeableness,^26^ our experimental results confirm that the default option can encourage EOL cancer patients to choose comfort‐oriented care goals. We do not propose any single type of EOL cancer care, but both comfort and LE options can be promoted through default options. When CC was assessed by health providers to be superior to LE treatments, CC goals could be preset to default in the ADs. There would need to be a careful balancing of the benefits and costs of aggressive treatments over comfort care, such as psychological stress, physical pain and financial burden. Given many patients' stated preferences for LE‐oriented care were the lack of knowledge about CC, such as hospice care, providing information on palliative care expands the informed choices of cancer patients. Providing information to EOL cancer patients on palliative care allows patients to make informed choices about ways of controlling the disease's symptoms and EOL quality of life. There may be superior clinical outcomes from comfort care. A study evaluating a representative sample of all deaths in America in 2004 found that palliative care achieves far better clinical outcomes than life‐extending treatments.^27^ In China, there is an urgent need to take measures to improve the population's awareness of CC. Further, any increased acceptance of CC would require the construction of new palliative care units and the strengthening of publicity about hospice care. To address any increase in comfort care choices by EOL cancer patients, Chinese health authorities might consider using medical insurance to encourage acceptance of CC, such as in South Korea where national medical insurance coverage directs EOL patients to comfort‐care cancer treatments.^27^

In previous research, order effects influenced decisions in domains as diverse as consumer preferences,^28^, ^29^ quality‐of‐life,^30^ and personality impression.^31^ However, order effects have been proven to be highly unstable in previous studies and to have little effect partly due to survey format,^32^ reference effect,^30^ familiarity and sensitivity.^29^ The influence of the first‐order effect was much weaker than the default option in our study. In the two standard AD groups, the order effect only showed significant differences in two individual‐specific palliative care options. We infer that for advanced cancer patients whose goal is to prolong life, the default option can affect their preferences, but the order effect is not obvious.

Our study also found that most patients chose to appoint their children to make EOL care decisions. 118 (65.9%) of our advanced cancer patients handed their agency to their children, not their spouses or siblings. The finding was different from Harvey's research, which reported that 94% of patients who were married or in a long‐term relationship selected their spouse or partner as the preferred decision‐maker in the eastern United States.^33^ This may be because China's traditional family culture attributes adult children as key members of the family with obligations to care for their parents.^34^

In addition, considering the fact that Chinese patients and their family members focused more on the life‐extending than the comfort‐care option, this higher level of direction by comfort‐care patients might support Chinese health providers in encouraging patients and their families to consider, where appropriate, CC through public advocacy and shared decision‐making, especially for those who might otherwise prioritize life‐extending care.

## 5 LIMITATIONS

Our study has several limitations. First, the sample enrolled a relatively small number of Chinese cancer patients from a single health system. Although it was a randomized survey, the sample size does not allow us to rule out the possibility that results were confounded by unmeasured variables, such as how well cancer patients understood their illnesses or how often they spoke with their physicians about their prognosis. Second, although we have built a scenario of ADs for patients with advanced cancer, due to real treatment restrictions, such as the Chinese medical act, the choices cancer patients made in our study were not always available for treatment. We note that comfort care poses problems when cancer responses may vary according to individual health issues, which is attenuated by our selection criteria, especially those aged over 40, stage III or IV cancer and hospitalized and receiving treatment. For future research, we recommend extending the selection criteria to include samples of other cancer patients.

## 6 CONCLUSION

In our study, patients with advanced cancer did not hold deep‐seated preferences for EOL care. Default options had a significant influence on patients' preferences for EOL care, while the order effect was significant only in specific palliative care treatment objectives. Therefore, the structure of ADs mattered and influenced the choice of different treatment outcomes, including the role of palliative care. While CC is more conducive to improving the quality of EOL care, the Chinese population's preference for CC was low, which makes it vital to strengthen the publicity and education around CC, including palliative care. Such an education and information campaign should be directed at both patients and patients' families, especially among children of patients who prefer LE care.

## AUTHOR CONTRIBUTIONS

Zi‐Meng Ye contributed towards the article by undertaking the statistical analysis and writing the manuscript. An‐Li Leng, Ben Ma and Jian Wang contributed towards the article by making substantial contributions to the conception, design, and interpretation of the data. An‐Li Leng, Stephen Nicholas and Elizabeth Maitland engaged in interpreting the results and writing the paper. All authors read and approved the final version of the manuscript.

## CONFLICT OF INTEREST STATEMENT

The authors declare no conflict of interest

## Supporting information

Supporting information.

Supporting information.

## Data Availability

The datasets used during the current study are available from the corresponding author.

## References

[1] Leng A , Jing J , Nicholas S , Wang J . Geographical disparities in treatment and health care costs for end‐of‐life cancer patients in China: a retrospective study. BMC Cancer. 2019;19(1):39. 10.1186/s12885-018-5237-1 30621633 PMC6325809

[2] Fried TR , Bradley EH , Towle VR , Allore H . Understanding the treatment preferences of seriously ill patients. N Engl J Med. 2002;346(14):1061‐1066. 10.1056/NEJMsa012528 11932474

[3] Laryionava K , Pfeil TA , Dietrich M , Reiter‐Theil S , Hiddemann W , Winkler EC . The second patient? Family members of cancer patients and their role in end‐of‐life decision making. BMC Palliat Care. 2018;17(1):29. 10.1186/s12904-018-0288-2 29454337 PMC5816525

[4] Hsu C‐Y , O'Connor M , Lee S . Understandings of death and dying for people of Chinese origin. Death Stud. 2009;33(2):153‐174. 10.1080/07481180802440431 19143109

[5] Agarwal R , Epstein AS . Advance care planning and end‐of‐life decision making for patients with cancer. Semin Oncol Nurs. 2018;34(3):316‐326. 10.1016/j.soncn.2018.06.012 30100366 PMC6156999

[6] Hinderer KA , Lee MC . Chinese Americans' attitudes toward advance directives: an assessment of outcomes based on a nursing‐led intervention. Appl Nurs Res. 2019;49:91‐96. 10.1016/j.apnr.2019.04.00 31160144

[7] Sutter R , Meyer‐Zehnder B , Baumann SM , Marsch S , Pargger H . Advance directives in the neurocritically ill: a systematic review. Crit Care Med. 2020;48(8):1188‐1195. 10.1097/CCM.0000000000004388 32697490

[8] Porteri C . Advance directives as a tool to respect patients' values and preferences: discussion on the case of Alzheimer's disease. BMC Med Ethics. 2018;19(1):9. 10.1186/s12910-018-0249-6 29458429 PMC5819243

[9] Fried TR . Garnering support for advance care planning. JAMA. 2010;303(3):269‐270. 10.1001/jama.2009.1956 20085956 PMC2899482

[10] Choudhry NK , Ma J , Rasooly I , Singer PA . Long‐term care facility policies on life‐sustaining treatments and advance directives in Canada. J Am Geriatr Soc. 1994;42:1150‐1153. 10.1111/j.1532-5415.1994.tb06980.x 7963200

[11] Veshi D , Neitzke G . Advance directives in some Western European countries: a legal and ethical comparison between Spain, France, England, and Germany. Eur J Health Law. 2015;22(4):321‐345. 10.1163/15718093-12341368 26427271

[12] Centers for Medicare & Medicaid Services . Physician fee schedule final rule with comment period. Accessed July 2. Accessed July 2,2018. https://www.cms.gov/Medicare/Medicare-Fee-for-Service-Payment/PhysicianFeeSched/PFS-Federal-Regulation-Notices-Items/CMS-1631-FC.html

[13] Degenholtz HB , Rhee Y , Arnold RM . Brief communication: the relationship between having a living will and dying in place. Ann Intern Med. 2004;141(2):113‐117. 10.7326/0003-4819-141-2-200407200-00009 15262666

[14] Silveira MJ , Kim SYH , Langa KM . Advance directives and outcomes of surrogate decision making before death. N Engl J Med. 2010;362(13):1211‐1218. 10.1056/NEJMc1005312 20357283 PMC2880881

[15] Halpern SD , Loewenstein G , Volpp KG , et al. Default options in advance directives influence how patients set goals for end‐of‐life care. Health Aff. 2013;32(2):408‐417. 10.1377/hlthaff.2012.0895 PMC4445426 23381535

[16] Halpern SD , Small DS , Troxel AB , et al. Effect of default options in advance directives on hospital‐free days and care choices among seriously ill patients: a randomized clinical trial. JAMA Netw open. 2020;3(3):e201742. 10.1001/jamanetworkopen.2020.1742 32227179 PMC7315782

[17] Halpern SD . Toward evidence‐based end‐of‐life care. N Engl J Med. 2015;373(21):2001‐2003. 10.1056/NEJMp1509664 26465826

[18] Yun YH , Kwak M , Park SM , et al. Chemotherapy use and associated factors among cancer patients near the end of life. Oncology. 2007;72(3‐4):164‐171. 10.1159/000112802 18097167

[19] Bansback N , Li LC , Lynd L , Bryan S . Exploiting order effects to improve the quality of decisions. Patient Educ Couns. 2014;96(2):197‐203. 10.1016/j.pec.2014.05.021 24961445

[20] Liao YH , Li CI , Lin CC , Lin JG , Chiang JH , Li TC . Traditional Chinese medicine as adjunctive therapy improves the long‐term survival of lung cancer patients. J Cancer Res Clin Oncol. 2017;143:2425‐2435. 10.1007/s00432-017-2491-6 28803328 PMC11819392

[21] Connors AF , Dawson NV , Desbiens NA , et al. A controlled trial to improve care for seriously III hospitalized patients: the study to understand prognoses and preferences for outcomes and risks of treatments (SUPPORT). JAMA. 1995;274(20):1591‐1598. 10.1001/jama.1995.03530200027032 7474243

[22] Johnson EJ , Hershey J , Meszaros J , Kunreuther H . Framing, probability distortions, and insurance decisions. J Risk Uncertain. 1993;7:35‐51.

[23] Beshears JL , Choi JJ , Laibson D , et al. The importance of default options for retirement saving outcomes: evidence from the United States. In: Brown J , Liebman J , Wise DA , eds. Social Security Policy in a Changing Environment. University of Chicago Press; 2011:165‐169.

[24] Madrian BC , Shea DF . The power of suggestion: inertia in 401(k) participation and savings behavior. Q J Econ. 2001;116(4):1149‐1187. 10.1162/003355301753265543

[25] Chapman GB , Li M , Colby H , Yoon H . Opting in vs. opting out of influenza vaccination. JAMA. 2010;304(1):43‐44. 10.1001/jama.2010.892 20606147

[26] Johnson EJ , Goldstein D . Do defaults save lives. Science. 2003;302(5649):1338‐1339. 10.1126/science.1091721 14631022

[27] Teno JM , Clarridge BR , Casey V , et al. Family perspectives on end‐of‐life care at the last place of care. JAMA. 2004;291(1):88‐93. 10.1001/jama.291.1.88 14709580

[28] Loginova O . Exposure order effects and advertising competition. J Econ Behav Organ. 2009;71(2):528‐538. 10.1016/j.jebo.2009.04.012

[29] Cao Y , Cranfield J , Widowski T . Position‐dependent order effects on the prediction of consumer preferences in repeated choice experiments. Appl Econ. 2018;50(3):287‐302. 10.1080/00036846.2017.1321836

[30] Day B , Bateman IJ , Carson RT , et al. Ordering effects and choice set awareness in repeat‐response stated preference studies. J Environ Econ Manage. 2012;63(1):73‐91. 10.1016/j.jeem.2011.09.001

[31] Norman R , Kemmler G , Viney R , et al. Order of presentation of dimensions does not systematically bias utility weights from a discrete choice experiment. Value Health. 2016;19:1033‐1038. 10.1016/j.jval.2016.07.003 27987630

[32] Anderson NH . Primacy effects in personality impression formation. J Pers Soc Psychol. 1965;2(1):1‐9. 10.1037/h0021966 14313838

[33] Harvey SV , Adenwala AY , Lane‐Fall MB . Defining familial interactions and networks: an exploratory qualitative study on family networks and surrogate decision‐making. Crit Care Explor. 2021;3(8):e0504. 34345829 10.1097/CCE.0000000000000504 PMC8323795

[34] Dai B , Mao Z , Wu B , Mei YJ , Levkoff S , Wang H . Family caregiver's perception of Alzheimer's disease and caregiving in Chinese culture. Soc Work Public Health. 2015;30(2):185‐196. 10.1080/19371918.2014.969858 25602761

