# Supplementary material for: Structuring healthcare advance directives: Evidence from Chinese end‐of‐life cancer patients' treatment preferences
Source: Health Expect. 2023 Apr 27;26(4):1648–57. doi: 10.1111/hex.13769 (PMC10349230; doi:10.1111/hex.13769)
Supplement: Supplementary file 1 — Supporting information. [file HEX-26--s001.docx]

### Appendix1

### Advance Directive Forms

**(Comfort default advance directive )**

**Part I – Durable Healthcare Power of Attorney**

I appoint the following agent __________________________(Name and relationship such as：spouse child parents or brothers and sisters) to be my agent to make health and personal care decisions for me **when and only when I lack sufficient capacity to make or communicate a choice regarding a health or personal care decision** as verified by my attending physician. My agent may not delegate the authority to make decisions.

You are not required to appoint an agent. If you don’t wish to appoint an agent, write “None” in the above space. If you do not name an agent, health care providers will ask your family for help in determining your wishes for treatment.

### Part II - Healthcare Treatment Instructions (Living Will)

The following health care treatment instructions exercise my right to make my own health care decisions. These instructions are intended to provide clear and convincing evidence of my wishes to be followed when I lack the capacity to understand, make, or communicate my treatment decisions. This section of the document will take effect **when and only when I lack the ability to understand, make or communicate a choice regarding a health or personal care decision as verified by my attending physician**.

### Overall Goals of Care

___X I want my health care providers and agent to treat me by helping relieve my pain and suffering,

even if that means that I may not live as long.

If you would prefer to choose a different option, **cross out** the lines above and place your initials beside one of the other options below:

_______ I want my health care providers and agent to treat me by helping me to live as long as possible, even if that means that I may suffer more pain or suffering.

## OR

_______ I do not want to specify one of the above goals. My health care providers and agent may direct the overall goals of my care.

In addition, I want my health care providers and agent to focus on the following goals (optional):

_____________________________________________________________________________________

_____________________________________________________________________________________

_____________________________________________________________________________________

### Specific Procedures

I request the specified for the following life prolonging procedures:

1. Cardiopulmonary resuscitation (CPR)

___X__ I do not want cardiopulmonary resuscitation (CPR) to be performed on me if my heart stops beating, even if performing CPR might prolong my life.

If you would prefer to choose a different option, **cross out** the lines above and place your initials beside one of the options below:

______ I request cardiopulmonary resuscitation (CPR) if my heart stops beating, even if performing CPR might increase my pain or suffering.

## OR

______ I do not wish to specify one of these options. My health care providers and agent may make any decisions about cardiopulmonary resuscitation (CPR) for me.

1. Intensive care unit (ICU) admission

___X__ I do not want to be admitted to the intensive care unit (ICU), even if it may prolong my life.

If you would prefer to choose a different option, **cross out** the lines above and place your initials beside one of the options below:

______ I want to be admitted to the intensive care unit (ICU) if it might prolong my life, even if it may increase my pain or suffering.

## OR

______ I do not wish to specify one of these options. My health care providers and agent may make any decisions about intensive care unit (ICU) admission for me.

1. Mechanical ventilator use

___X__ I do not want a mechanical ventilator to be used if it is needed to prolong my life. In that case, I would prefer care directed towards relief of pain and suffering.

If you would prefer to choose a different option, **cross out** the lines above and place your initials beside one of the options below:

______ I request the use of a mechanical ventilator if it may prolong my life, even if it may also increase my pain or suffering.

## OR

______ I do not wish to specify one of these options. My health care providers and agent may

make any decisions about mechanical ventilator use for me.

1. Dialysis (kidney filtration by machine)

___X__ I do not want dialysis to be performed on me, even if it might prolong my life.

If you would prefer to choose a different option, **cross out** the lines above and place your initials beside one of the options below:

______ I request dialysis if it may prolong my life, even if it may also increase my pain or suffering.

## OR

______ I do not wish to specify one of these options. My health care providers and agent may make any decisions about the use of dialysis for me.

1. Feeding tube insertion

___X__ I do not want to have a feeding tube inserted, even if it might prolong my life.

If you would prefer to choose a different option, **cross out** the lines above and place your initials beside one of the options below:

______ I request feeding tube insertion if it may prolong my life, even if it may also increase my pain or suffering.

## OR

______ I do not wish to specify one of these options. My health care providers and agent may make any decisions about feeding tube for me.

1. Surgery

___X__ I do not want to accept surgery, even if it might prolong my life.

If you would prefer to choose a different option, **cross out** the lines above and place your initials beside one of the options below:

______ I want to accept surgery if it may prolong my life, even if it may also increase my pain or suffering.

## OR

______ I do not wish to specify one of these options. My health care providers and agent may make any decisions about surgery tube for me.

1. Radiotherapy

___X__ I do not want to accept radiotherapy, even if it might prolong my life.

If you would prefer to choose a different option, **cross out** the lines above and place your initials beside one of the options below:

______ I request to accept radiotherapy if it may prolong my life, even if it may also increase my pain or suffering.

## OR

______ I do not wish to specify one of these options. My health care providers and agent may make any decisions about radiotherapy for me.

1. Chemotherapy

___X__ I do not want to accept chemotherapy, even if it might prolong my life.

If you would prefer to choose a different option, **cross out** the lines above and place your initials beside one of the options below:

______ I request to accept chemotherapy if it may prolong my life, even if it may also increase my pain or suffering.

## OR

______ I do not wish to specify one of these options. My health care providers and agent may make any decisions about chemotherapy for me.

1. Palliative care ($454.month)

A caring hospital provides palliative treatment, which can reduce pain, relieve depression and anxiety and other nursing services, and provide accommodation. The patients will be looked after by special doctors and nurses, and the family members do not need to accompany them. The cost is 3000 yuan ($454) a month.

___X__I asked for palliative care if it would relieve the pain and burden on my family, even though it might not prolong my life.

If you would prefer to choose a different option, **cross out** the lines above and place your initials beside one of the options below:

______I do not wish to receive palliative care, even though it may reduce the pain and burden on my family.

## OR

______ I do not wish to specify one of these options. My health care providers and agent may make any decisions about palliative care for me.

1. Palliative care ($1059/month)

A caring hospital provides palliative treatment, which can reduce pain, relieve depression and anxiety and other nursing services, and provide accommodation. The patients will be looked after by special doctors and nurses, and the family members do not need to accompany them. The cost is 7000 yuan ($1059) a month.

___X__I asked for palliative care if it would relieve the pain and burden on my family, even though it might not prolong my life.

If you would prefer to choose a different option, **cross out** the lines above and place your initials beside one of the options below:

______ I do not wish to receive palliative care, even though it may reduce the pain and burden on my family.

## OR

______ I do not wish to specify one of these options. My health care providers and agent may make any decisions about palliative care for me.

1. Traditional Chinese medicine treatment

___X__ I do not want to accept traditional Chinese medicine treatment, even if it might prolong my life.

If you would prefer to choose a different option, **cross out** the lines above and place your initials beside one of the options below:

______ I request accept traditional Chinese medicine treatment if it may prolong my life, even if it may also increase my pain or suffering.

## OR

______ I do not wish to specify one of these options. My health care providers and agent may make any decisions about traditional Chinese medicine treatment for me.

### Agent’s Use of Instructions (Initial one option only)

___________ My agent must follow these instructions.

## OR

___________ These instructions are only guidance. My agent shall have final say, and may override any of my instructions unless I specifically indicate exceptions.

If I did not appoint an agent, these instructions shall be followed.

### Standard Advance Directive Forms

**(Standard comfort-oriented care)**

**Part I – Durable Healthcare Power of Attorney**

I appoint the following agent __________________________(Name and relationship such as：spouse child parents or brothers and sisters) to be my agent to make health and personal care decisions for me **when and only when I lack sufficient capacity to make or communicate a choice regarding a health or personal care decision** as verified by my attending physician. My agent may not delegate the authority to make decisions.

You are not required to appoint an agent. If you don’t wish to appoint an agent, write “None” in the above space. If you do not name an agent, health care providers will ask your family for help in determining your wishes for treatment.

### Part II - Healthcare Treatment Instructions (Living Will)

The following health care treatment instructions exercise my right to make my own health care decisions. These instructions are intended to provide clear and convincing evidence of my wishes to be followed when I lack the capacity to understand, make, or communicate my treatment decisions. This section of the document will take effect **when and only when I lack the ability to understand, make or communicate a choice regarding a health or personal care decision as verified by my attending physician**.

### Overall Goals of Care

___ I want my health care providers and agent to treat me by helping relieve my pain and suffering,

even if that means that I may not live as long.

## OR

_______ I want my health care providers and agent to treat me by helping me to live as long as possible, even if that means that I may suffer more pain or suffering.

## OR

_______ I do not want to specify one of the above goals. My health care providers and agent may direct the overall goals of my care.

In addition, I want my health care providers and agent to focus on the following goals (optional):

_____________________________________________________________________________________

_____________________________________________________________________________________

_____________________________________________________________________________________

### Specific Procedures

I request the specified for the following life prolonging procedures:

1. Cardiopulmonary resuscitation (CPR)

______ I do not want cardiopulmonary resuscitation (CPR) to be performed on me if my heart stops beating, even if performing CPR might prolong my life.

## OR

______ I request cardiopulmonary resuscitation (CPR) if my heart stops beating, even if performing CPR might increase my pain or suffering.

## OR

______ I do not wish to specify one of these options. My health care providers and agent may make any decisions about cardiopulmonary resuscitation (CPR) for me.

1. Intensive care unit (ICU) admission

______ I do not want to be admitted to the intensive care unit (ICU), even if it may prolong my life.

## OR

______ I want to be admitted to the intensive care unit (ICU) if it might prolong my life, even if it may increase my pain or suffering.

## OR

______ I do not wish to specify one of these options. My health care providers and agent may make any decisions about intensive care unit (ICU) admission for me.

1. Mechanical ventilator use

______ I do not want a mechanical ventilator to be used if it is needed to prolong my life. In that case, I would prefer care directed towards relief of pain and suffering.

## OR

______ I request the use of a mechanical ventilator if it may prolong my life, even if it may also increase my pain or suffering.

## OR

______ I do not wish to specify one of these options. My health care providers and agent may

make any decisions about mechanical ventilator use for me.

1. Dialysis (kidney filtration by machine)

______ I do not want dialysis to be performed on me, even if it might prolong my life.

## OR

______ I request dialysis if it may prolong my life, even if it may also increase my pain or suffering.

## OR

______ I do not wish to specify one of these options. My health care providers and agent may make any decisions about the use of dialysis for me.

1. Feeding tube insertion

______ I do not want to have a feeding tube inserted, even if it might prolong my life.

## OR

______ I request feeding tube insertion if it may prolong my life, even if it may also increase my pain or suffering.

## OR

______ I do not wish to specify one of these options. My health care providers and agent may make any decisions about feeding tube for me.

1. Surgery

______ I do not want to accept surgery, even if it might prolong my life.

## OR

______ I want to accept surgery if it may prolong my life, even if it may also increase my pain or suffering.

## OR

______ I do not wish to specify one of these options. My health care providers and agent may make any decisions about surgery for me.

1. Radiotherapy

______ I do not want to accept radiotherapy, even if it might prolong my life.

## OR

______ I request to accept radiotherapy if it may prolong my life, even if it may also increase my pain or suffering.

## OR

______ I do not wish to specify one of these options. My health care providers and agent may make any decisions about radiotherapy for me.

1. Chemotherapy

______ I do not want to accept chemotherapy, even if it might prolong my life.

## OR

______ I request to accept chemotherapy if it may prolong my life, even if it may also increase my pain or suffering.

## OR

______ I do not wish to specify one of these options. My health care providers and agent may make any decisions about chemotherapy for me.

1. Palliative care ($454/month)

A caring hospital provides palliative treatment, which can reduce pain, relieve depression and anxiety and other nursing services, and provide accommodation. The patients will be looked after by special doctors and nurses, and the family members do not need to accompany them. The cost is 3000 yuan ($454) a month.

______I asked for palliative care if it would relieve the pain and burden on my family, even though it might not prolong my life..

## OR

______ I do not wish to receive palliative care, even though it may reduce the pain and burden on my family.

## OR

______ I do not wish to specify one of these options. My health care providers and agent may make any decisions about palliative care for me.

1. Palliative care ($1059/month)

A caring hospital provides palliative treatment, which can reduce pain, relieve depression and anxiety and other nursing services, and provide accommodation. The patients will be looked after by special doctors and nurses, and the family members do not need to accompany them. The cost is 7000 yuan ($454) a month.

______ I asked for palliative care if it would relieve the pain and burden on my family, even though it might not prolong my life.

## OR

______ I do not wish to receive palliative care, even though it may reduce the pain and burden on my family.

## OR

______ I do not wish to specify one of these options. My health care providers and agent may make any decisions about palliative care for me.

1. Traditional Chinese medicine treatment

______ I do not want to accept traditional Chinese medicine treatment, even if it might prolong my life.

## OR

______ I request accept traditional Chinese medicine treatment if it may prolong my life, even if it may also increase my pain or suffering.

## OR

______ I do not wish to specify one of these options. My health care providers and agent may make any decisions about traditional Chinese medicine treatment for me.

### Agent’s Use of Instructions (Initial one option only)

___________ My agent must follow these instructions.

## OR

___________ These instructions are only guidance. My agent shall have final say, and may override any of my instructions unless I specifically indicate exceptions.

If I did not appoint an agent, these instructions shall be followed.

### Advance Directive Forms

**(Life-extension default advance directive)**

### Part I – Durable Healthcare Power of Attorney

I appoint the following agent __________________________(Name and relationship such as: spouse child parents or brothers and sisters) to be my agent to make health and personal care decisions for me **when and only when I lack sufficient capacity to make or communicate a choice regarding a health or personal care decision** as verified by my attending physician. My agent may not delegate the authority to make decisions.

You are not required to appoint an agent. If you don’t wish to appoint an agent, write “None” in the above space. If you do not name an agent, health care providers will ask your family for help in determining your wishes for treatment.

### Part II - Healthcare Treatment Instructions (Living Will)

The following health care treatment instructions exercise my right to make my own health care decisions. These instructions are intended to provide clear and convincing evidence of my wishes to be followed when I lack the capacity to understand, make, or communicate my treatment decisions. This section of the document will take effect **when and only when I lack the ability to understand, make or communicate a choice regarding a health or personal care decision as verified by my attending physician**.

### Overall Goals of Care

___X I want my health care providers and agent to treat me by helping me to live as long as possible,

even if that means that I may have more pain or suffering.

If you would prefer to choose a different overall goal of care, **cross out** the lines above and place your initials beside one of the other options below:

_______ I want my health care providers and agent to treat me by helping relieve my pain and suffering, even if that means that I may not live as long.

## OR

_______ I do not want to specify one of the above goals. My health care providers and agent may direct the overall goals of my care.

In addition, I want my health care providers and agent to focus on the following goals (optional):

_____________________________________________________________________________________

_____________________________________________________________________________________

_____________________________________________________________________________________

### Specific Procedures

I make the following specific requests regarding life- prolonging procedures:

1. Cardiopulmonary resuscitation (CPR)

___X__ I request cardiopulmonary resuscitation (CPR) if my heart stops beating, even if performing CPR may increase my pain or suffering.

If you would prefer to choose a different option, **cross out** the lines above and place your initials beside one of the options below:

______ I do not want cardiopulmonary resuscitation (CPR) to be performed on me if my heart stops beating, even if performing CPR might prolong my life.

## OR

______ I do not wish to specify one of these options. My health care providers and agent may make any decisions about cardiopulmonary resuscitation (CPR) for me.

1. Intensive care unit (ICU) admission

___X__ I want to be admitted to the intensive care unit (ICU) if it may prolong my life, even if it may increase my pain or suffering.

If you would prefer to choose a different option, **cross out** the lines above and place your initials beside one of the options below:

______ I do not want to be admitted to the intensive care unit (ICU), even if it might prolong my life.

## OR

______ I do not wish to specify one of these options. My health care providers and agent may make any decisions about intensive care unit (ICU) admission for me.

1. Mechanical ventilator use

___X__ I request the use of a mechanical ventilator if it may prolong my life, even if it may also increase my pain or suffering.

If you would prefer to choose a different option, **cross out** the lines above and place your initials beside one of the options below:

______ I do not want a mechanical ventilator to be used if it is needed to prolong my life. In that case, I would prefer care directed towards relief of pain and suffering.

## OR

______ I do not wish to specify one of these options. My health care providers and agent may make any decisions about mechanical ventilator use for me.

1. Dialysis (kidney filtration by machine)

___X__ I request dialysis if it may prolong my life, even if it may also increase my pain or suffering.

If you would prefer to choose a different option, **cross out** the lines above and place your initials beside one of the options below:

______ I do not want dialysis to be performed on me, even if it might prolong my life.

## OR

______ I do not wish to specify one of these options. My health care providers and agent may make any decisions about the use of dialysis for me.

1. Feeding tube insertion

___X__ I request feeding tube insertion if it may prolong my life, even if it may also increase my pain or suffering.

If you would prefer to choose a different option, **cross out** the lines above and place your initials beside one of the options below:

______ I do not want to have a feeding tube inserted, even if it might prolong my life.

## OR

______ I do not wish to specify one of these options. My health care providers and agent may make any decisions about feeding tube for me.

1. Surgery

___X__ I request accept surgery if it may prolong my life, even if it may also increase my pain or suffering.

If you would prefer to choose a different option, **cross out** the lines above and place your initials beside one of the options below:

______ I do not want to accept surgery, even if it might prolong my life.

## OR

______ I do not wish to specify one of these options. My health care providers and agent may make any decisions about surgery for me.

1. Radiotherapy

___X__ I want to accept radiotherapy if it may prolong my life, even if it may also increase my pain or suffering.

If you would prefer to choose a different option, **cross out** the lines above and place your initials beside one of the options below:

______ I do not want to accept radiotherapy, even if it might prolong my life.

## OR

______ I do not wish to specify one of these options. My health care providers and agent may make any decisions about radiotherapy for me.

1. Chemotherapy

___X__ I request chemotherapy if it may prolong my life, even if it may also increase my pain or suffering.

If you would prefer to choose a different option, **cross out** the lines above and place your initials beside one of the options below:

______ I do not want to accept chemotherapy, even if it might prolong my life.

## OR

______ I do not wish to specify one of these options. My health care providers and agent may make any decisions about chemotherapy for me.

1. Palliative care ($454/month)

A caring hospital provides palliative treatment, which can reduce pain, relieve depression and anxiety and other nursing services, and provide accommodation. The patients will be looked after by special doctors and nurses, and the family members do not need to accompany them. The cost is 3000 yuan ($454) a month.

___X__ I do not wish to receive palliative care, even though it may reduce the pain and burden on my family.

If you would prefer to choose a different option, **cross out** the lines above and place your initials beside one of the options below:

______ I asked for palliative care if it would relieve the pain and burden on my family, even though it might not prolong my life.

## OR

______ I do not wish to specify one of these options. My health care providers and agent may make any decisions about palliative care for me.

1. Palliative care ($1059/month)

A caring hospital provides palliative treatment, which can reduce pain, relieve depression and anxiety and other nursing services, and provide accommodation. The patients will be looked after by special doctors and nurses, and the family members do not need to accompany them. The cost is 7000 yuan ($1059) a month.

___X__I do not wish to receive palliative care, even though it may reduce the pain and burden on my family.

If you would prefer to choose a different option, **cross out** the lines above and place your initials beside one of the options below:

______ I asked for palliative care if it would relieve the pain and burden on my family, even though it might not prolong my life.

## OR

______ I do not wish to specify one of these options. My health care providers and agent may make any decisions about palliative care for me.

1. Traditional Chinese medicine treatment

___X__ I request traditional Chinese medicine treatment if it may prolong my life, even if it may also increase my pain or suffering.

If you would prefer to choose a different option, **cross out** the lines above and place your initials beside one of the options below:

______ I do not want to have traditional Chinese medicine treatment, even if it might prolong my life.

## OR

______ I do not wish to specify one of these options. My health care providers and agent may make any decisions about traditional Chinese medicine treatment for me.

### Agent’s Use of Instructions (Initial one option only)

___________ My agent must follow these instructions.

## OR

___________ These instructions are only guidance. My agent shall have final say, and may override any of my instructions unless I specifically indicate exceptions.

If I did not appoint an agent, these instructions shall be followed.

**(Standard Advance Directive Forms**

**(Standard life extension-oriented care)**

### Part I – Durable Healthcare Power of Attorney

I appoint the following agent __________________________ (Name and relationship such as: spouse child parents or brothers and sisters) to be my agent to make health and personal care decisions for me **when and only when I lack sufficient capacity to make or communicate a choice regarding a health or personal care decision** as verified by my attending physician. My agent may not delegate the authority to make decisions.

You are not required to appoint an agent. If you don’t wish to appoint an agent, write “None” in the above space. If you do not name an agent, health care providers will ask your family for help in determining your wishes for treatment.

### Part II - Healthcare Treatment Instructions (Living Will)

The following health care treatment instructions exercise my right to make my own health care decisions. These instructions are intended to provide clear and convincing evidence of my wishes to be followed when I lack the capacity to understand, make, or communicate my treatment decisions. This section of the document will take effect **when and only when I lack the ability to understand, make or communicate a choice regarding a health or personal care decision as verified by my attending physician**.

### Overall Goals of Care

___ I want my health care providers and agent to treat me by helping me to live as long as possible,

even if that means that I may have more pain or suffering.

## OR

_______ I want my health care providers and agent to treat me by helping relieve my pain and suffering, even if that means that I may not live as long.

## OR

_______ I do not want to specify one of the above goals. My health care providers and agent may direct the overall goals of my care.

In addition, I want my health care providers and agent to focus on the following goals (optional):

_____________________________________________________________________________________

_____________________________________________________________________________________

_____________________________________________________________________________________

### Specific Procedures

I make the following specific requests regarding life- prolonging procedures:

1. Cardiopulmonary resuscitation (CPR)

______ I request cardiopulmonary resuscitation (CPR) if my heart stops beating, even if performing CPR may increase my pain or suffering.

## OR

______ I do not want cardiopulmonary resuscitation (CPR) to be performed on me if my heart stops beating, even if performing CPR might prolong my life.

## OR

______ I do not wish to specify one of these options. My health care providers and agent may make any decisions about cardiopulmonary resuscitation (CPR) for me.

1. Intensive care unit (ICU) admission

______ I want to be admitted to the intensive care unit (ICU) if it may prolong my life, even if it may increase my pain or suffering.

## OR

______ I do not want to be admitted to the intensive care unit (ICU), even if it might prolong my life.

## OR

______ I do not wish to specify one of these options. My health care providers and agent may make any decisions about intensive care unit (ICU) admission for me.

1. Mechanical ventilator use

______ I request the use of a mechanical ventilator if it may prolong my life, even if it may also increase my pain or suffering.

## OR

______ I do not want a mechanical ventilator to be used if it is needed to prolong my life. In that case, I would prefer care directed towards relief of pain and suffering.

## OR

______ I do not wish to specify one of these options. My health care providers and agent may make any decisions about mechanical ventilator use for me.

1. Dialysis (kidney filtration by machine)

______ I request dialysis if it may prolong my life, even if it may also increase my pain or suffering.

## OR

______ I do not want dialysis to be performed on me, even if it might prolong my life.

## OR

______ I do not wish to specify one of these options. My health care providers and agent may make any decisions about the use of dialysis for me.

1. Feeding tube insertion

______ I request feeding tube insertion if it may prolong my life, even if it may also increase my pain or suffering.

## OR

______ I do not want to have a feeding tube inserted, even if it might prolong my life.

## OR

______ I do not wish to specify one of these options. My health care providers and agent may make any decisions about feeding tube for me.

1. Surgery

______ I request accept surgery if it may prolong my life, even if it may also increase my pain or suffering.

## OR

______ I do not want to accept surgery, even if it might prolong my life.

## OR

______ I do not wish to specify one of these options. My health care providers and agent may make any decisions about surgery for me.

1. Radiotherapy

______ I want to accept radiotherapy if it may prolong my life, even if it may also increase my pain or suffering.

## OR

______ I do not want to accept radiotherapy, even if it might prolong my life.

## OR

______ I do not wish to specify one of these options. My health care providers and agent may make any decisions about radiotherapy for me.

1. Chemotherapy

______ I request chemotherapy if it may prolong my life, even if it may also increase my pain or suffering.

## OR

______ I do not want to accept chemotherapy, even if it might prolong my life.

## OR

______ I do not wish to specify one of these options. My health care providers and agent may make any decisions about chemotherapy for me.

1. Palliative care ($454/month)

A caring hospital provides palliative treatment, which can reduce pain, relieve depression and anxiety and other nursing services, and provide accommodation. The patients will be looked after by special doctors and nurses, and the family members do not need to accompany them. The cost is 3000 yuan ($454) a month.

______ I do not wish to receive palliative care, even though it may reduce the pain and burden on my family.

## OR

______ I asked for palliative care if it would relieve the pain and burden on my family, even though it might not prolong my life.

## OR

______ I do not wish to specify one of these options. My health care providers and agent may make any decisions about palliative care for me.

1. Palliative care ($1059/month)

A caring hospital provides palliative treatment, which can reduce pain, relieve depression and anxiety and other nursing services, and provide accommodation. The patients will be looked after by special doctors and nurses, and the family members do not need to accompany them. The cost is 7000 yuan ($454) a month.

______I do not wish to receive palliative care, even though it may reduce the pain and burden on my family.

## OR

______ I asked for palliative care if it would relieve the pain and burden on my family, even though it might not prolong my life.

## OR

______ I do not wish to specify one of these options. My health care providers and agent may make any decisions about palliative care for me.

1. Traditional Chinese medicine treatment

______ I request traditional Chinese medicine treatment if it may prolong my life, even if it may also increase my pain or suffering.

## OR

______ I do not want to have traditional Chinese medicine treatment, even if it might prolong my life.

## OR

______ I do not wish to specify one of these options. My health care providers and agent may make any decisions about traditional Chinese medicine treatment for me.

### Agent’s Use of Instructions (Initial one option only)

___________ My agent must follow these instructions.

## OR

___________ These instructions are only guidance. My agent shall have final say, and may override any of my instructions unless I specifically indicate exceptions.

If I did not appoint an agent, these instructions shall be followed.
