# Supplementary material for: Structuring healthcare advance directives: Evidence from Chinese end‐of‐life cancer patients' treatment preferences
Source: Health Expect. 2023 Apr 27;26(4):1648–57. doi: 10.1111/hex.13769 (PMC10349230; doi:10.1111/hex.13769)
Supplement: Supplementary file 2 — Supporting information. [file HEX-26--s002.docx]

**Supplement**

| **eTable 1. Differences Among the 4 Versions of the Advance Directive** | | | | |
| --- | --- | --- | --- | --- |
|  | Comfort default ADs | Life-extension default ADs | Standard CC ADs | Standard LE ADs |
| Overall goal of care | Overall care focused on comfort-oriented care presented first and preselected  Overall care focused on comfort-oriented care and then preference not to choose presented next  Patient instructed to cross out preselected option and initial one of the other options if they prefer | Overall care focused on life-extension oriented care presented first and preselected  Overall care focused on life-extension oriented care and then preference not to choose presented next  Patient instructed to cross out preselected option and initial one of the other options if they prefer | Overall care focused on comfort option presented in first order, followed by preference not to choose  No option is preselected | Overall care focused on life-extension presented in first order followed by preference not to choose  No option is preselected |
| Specific objectives of care | Choices to forgo each of 11 life-support interventions presented first and preselected  Choices to receive each of 11 life-support interventions presented next  Patient instructed to cross out preselected option and initial one of the other options if they prefer | Choices to receive each of 11 life-support interventions presented first and preselected  Choices to forgo each of 11 life-support interventions presented next  Patient instructed to cross out preselected option and initial one of the other options if they prefer | Choices to forgo and receive each of 11 life-support interventions  Forgo option in first order presented, followed by preference not to choose  No options are preselected | Choices to receive and forgo each of 11 life-support interventions  Receive in first order presented, followed by preference not to choose  No options are preselected |

| **eTable 2. Comparison of Patients in advance directive group(n=179)** | | | | | |
| --- | --- | --- | --- | --- | --- |
| **Characteristic** | No. (%) | | | | **P-value** |
|  | **Comfort Default ADs (N=43)** | **Life-extension default ADs (N=50)** | **Standard** **CC ADs (N=43)** | **Standard LE ADs (N=43)** |  |
| Age, mean (SD) | 63.1 | 60.5 | 62.0 | 63.1 | **0.31** |
| **Sex** | | | | | **0.70** |
| Male | 32 (74.3) | 32 (64.0) | 31 (72.1) | 31 (72.1) |  |
| Female | 11 (25.6) | 18 (32.0) | 12 (27.9) | 12 (27.9) |  |
| **Marital status** | | | | | **0.79** |
| Married | 40 (93.0) | 45 (90.0) | 41 (95.4) | 42 (97.6) |  |
| Unmarried/widowed/divorced | 3 (7.0) | 4 (10.0） | 2 (4.6) | 1 (2.4) |  |
| **Current Cancer Stage** | | | | | **0.14** |
| Stage III | 27 (62.8) | 20 (40.0) | 20 (46.5) | 24 (55.8) |  |
| Stage IV | 16 (37.2) | 30 (60.0) | 23 (53.5) | 19 (44.2) |  |
| **Relation** | | | | | **0.92** |
| Spouse | 11(25.6) | 15(30.0) | 10(23.3) | 11(25.6) |  |
| Child | 28(65.1) | 28(56.0) | 31(72.1) | 31(72.1) |  |
| Parent | 2(4.7) | 3(6.0) | 0(0) | 0(0) |  |
| Brothers and sisters | 0(0) | 1(2.0) | 4(2.3) | 0(0) |  |
| None | 2(4.7) | 3(6.0) | 1(2.3) | 1(2.3) |  |
| **Agent Power** | | | | | **0.19** |
| Must follow patient's decision | 13(30.2) | 3(6.0) | 6(14.0) | 35(81.4) |  |
| Have the right to decide | 29(67.4) | 45(90.0) | 36(83.7) | 7(16.3) |  |
| Not selected | 1(2.3) | 2(4.0) | 1(2.3) | 1(2.3) |  |
| US$, United States dollars  a Based on a currency exchange rate of the 6.6118 yuan to US$1.00 in 2018. | | | | | |

| **eTable 3. Comparison of Patients with total goal and 11 life-support interventions in advance directive group（n=179）** | | | | | |
| --- | --- | --- | --- | --- | --- |
| **Characteristic** | No. (%) | | | | **P-value** |
|  | **Comfort Default ADs (N=43)** | **Life-extension default ADs (N=50)** | **Standard CC ADs (N=43)** | **Standard LE ADs (N=43)** |  |
| **Total goal** | | | | | 0.00 |
| Comfort-oriented care | 14(32.6) | 10(20.0) | 7(16.3) | 10(23.3) |  |
| Life extension-oriented care | 14(32.6) | 33(66.0) | 25(58.1) | 16(37.2) |  |
| Surrogates | 15(34.9) | 7(14.0) | 11(27.9) | 17(39.5) |  |
| **Cardiopulmonary resuscitation (CPR)** | | | | | 0.00 |
| Comfort-oriented care | 14(32.6) | 6(12.0) | 9(20.9) | 9(20.9) |  |
| Life extension-oriented care | 15(34.9) | 34(68.0) | 21(48.8) | 17(39.5) |  |
| Surrogates | 14(32.6) | 10(20.0) | 13(30.2) | 17(39.5) |  |
| **Intensive care unit (ICU) admission** | | | | | 0.00 |
| Comfort-oriented care | 13(30.2) | 10(20.0) | 7(16.3) | 12(27.9) |  |
| Life extension-oriented care | 12(27.9) | 31(62.0) | 24(55.8) | 13(30.2) |  |
| Surrogates | 18(41.86) | 9(18.0) | 12(27.9) | 18(41.86) |  |
| **Mechanical ventilator use** | | | | | 0.02 |
| Comfort-oriented care | 13(30.2) | 11(22.0) | 8(18.6) | 10(23.3) |  |
| Life extension-oriented care | 11(25.6) | 28(56.0) | 22(51.2) | 18(41.86) |  |
| Surrogates | 19(44.2) | 11(22.0) | 13(30.2) | 15(34.9) |  |
| **Dialysis (kidney filtration by machine)** | | | | | 0.02 |
| Comfort-oriented care | 11(27.9) | 8(16.0) | 9(20.9) | 9(20.9) |  |
| Life extension-oriented care | 16(37.2) | 30(60.0) | 23(53.5) | 16(37.2) |  |
| Surrogates | 16(37.2) | 12(24.0) | 11(27.9) | 18(41.86) |  |
| **Feeding tube insertion** | | | | | 0.00 |
| Comfort-oriented care | 9(20.9) | 8(16.0) | 7(16.3) | 13(30.2) |  |
| Life extension-oriented care | 16(37.2) | 32(64.0) | 22(51.2) | 13(30.2) |  |
| Surrogates | 18(41.86) | 10(20.0) | 14(32.6) | 17(39.5) |  |
| **Surgery** | | | | | 0.00 |
| Comfort-oriented care | 5(11.6) | 5(10.0) | 3(7.0) | 3(7.0) |  |
| Life extension-oriented care | 22(51.2) | 38(76.0) | 26(60.5) | 26(60.5) |  |
| Surrogates | 16(37.2) | 7(14.0) | 14(32.6) | 14(32.6) |  |
| **Radiotherapy** | | | | | 0.00 |
| Comfort-oriented care | 9(20.9) | 7(14.0) | 5(11.6) | 7(16.3) |  |
| Life extension-oriented care | 16(37.2) | 35(70.0) | 27(62.8) | 23(53.5) |  |
| Surrogates | 18(41.86) | 8(16.0) | 11(27.9) | 13(30.2) |  |
| **Chemotherapy** | | | | | 0.00 |
| Comfort-oriented care | 7(16.3) | 4(8.0) | 5(11.6) | 9(20.9) |  |
| Life extension-oriented care | 15(34.9) | 38(76.0) | 27(62.8) | 20(46.5) |  |
| Surrogates | 21(48.8) | 8(16.0) | 11(27.9) | 14(32.6) |  |
| **Palliative care ($454/month)** | | | | | 0.00 |
| Comfort-oriented care | 14(32.6) | 11(22.0) | 19(44.2) | 12(27.9) |  |
| Life extension-oriented care | 6(14.0) | 34(68.0) | 14(32.6) | 13(30.2) |  |
| Surrogates | 23(53.5) | 5(10.0) | 10(23.3) | 18(41.86) |  |
| **Palliative care ($1059/month)** | | | | | 0.00 |
| Comfort-oriented care | 12(27.9) | 8(16.0) | 14(32.6) | 10(23.3) |  |
| Life extension-oriented care | 9(20.9) | 37(74.0) | 20(46.5) | 14(32.6) |  |
| Surrogates | 22(51.2) | 5(10.0) | 9(20.9) | 19(44.2) |  |
| **Traditional Chinese medicine treatment** | | | | | 0.00 |
| Comfort-oriented care | 2(4.7) | 2(4.0) | 3(7.0) | 1(2.3) |  |
| Life extension-oriented care | 19(44.2) | 42(84.0) | 32(74.4) | 24(55.8) |  |
| Surrogates | 22(51.2) | 6(12.0) | 8(18.6) | 18(41.86) |  |

| **eTable 4. Comparison of Patients in advance directive group(T-test)** | | | | |
| --- | --- | --- | --- | --- |
|  | **Comfort care default Ads VS Life-extension default ADs** | **Standard CC ADs VS Standard LE ADs** | **Comfort care default Ads VS Standard CC ADs** | **Life-extension default Ads VS Standard LE ADs** |
| Agent power | 0.00 | 0.13 | 0.04 | 0.00 |
| Relation | 0.90 | 0.61 | 0.91 | 0.57 |
